# Enzymological and structural characterization of *Arabidopsis thaliana* heme oxygenase‐1

**DOI:** 10.1002/2211-5463.13453

**Published:** 2022-06-20

**Authors:** Jia Wang, Xiaoyi Li, Jing‐Wen Chang, Tong Ye, Ying Mao, Xiao Wang, Lin Liu

**Affiliations:** ^1^ School of Life Sciences Anhui University Hefei China; ^2^ Key Laboratory of Photobiology, Institute of Botany Chinese Academy of Sciences Beijing China

**Keywords:** *Arabidopsis thaliana*, biliverdin, crystallography, heme, heme oxygenase

## Abstract

*Arabidopsis thaliana* heme oxygenase‐1 (AtHO‐1), a metabolic enzyme in the heme degradation pathway, serves as a prototype for study of the bilin‐related functions in plants. Past biological analyses revealed that AtHO‐1 requires ferredoxin‐NADP^+^ reductase (FNR) and ferredoxin for its enzymatic activity. Here, we characterized the binding and degradation of heme by AtHO‐1, and found that ferredoxin is a dispensable component of the reducing system that provides electrons for heme oxidation. Furthermore, we reported the crystal structure of heme‐bound AtHO‐1, which demonstrates both conserved and previously undescribed features of plant heme oxygenases. Finally, the electron transfer pathway from FNR to AtHO‐1 is suggested based on the known structural information.

## Graphical abstract

Here, we found that heme oxygenase of *Arabidopsis thaliana* (AtHO‐1) can cleave heme in the absence of ferredoxin. Combined with the crystal structure of heme‐bound AtHO‐1, a shortcut for electron transfer from ferredoxin‐NADP^+^ reductase to AtHO‐1 was proposed.

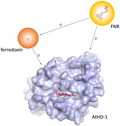

**Abbreviations**
CPR: NADPH‐cytochrome P450 reductase
DFO: deferoxamine
FNR: ferredoxin‐NADP^+^ reductase
HO: heme oxygenase
ITC: isothermal titration calorimetry
SEC: size‐exclusion chromatography

Heme oxygenases (HOs; EC1.14.14.18) catalyze oxidative conversion of heme to biliverdin and are widely distributed in plants, animals, and prokaryotes [1, 2]. Biliverdin is the universal precursor for plant phytochrome chromophore, animal bilirubin, and cyanobacterial phycobilins [3, 4, 5]. Heme oxidation catalyzed by HOs also produces iron ion and carbon monoxide (CO), and hence HOs play key roles in iron metabolism and CO signaling [6, 7]. Canonical HOs share a conserved HO fold primarily consisting of α‐helices. The model plant *Arabidopsis thaliana* has four HOs (AtHO‐1–AtHO‐4), which are clustered into HO1 subfamily (AtHO‐1, AtHO‐3, and AtHO‐4) and HO2 subfamily (AtHO‐2) [8, 9]. The main differences of HO2 from HO1 are the replacement of the ligating His with Arg and the presence of a spacer sequence that is rich in Glu and Asp residues. An *in vitro* study of AtHO‐1–AtHO‐4 has shown that the HO1 subfamily members are authentic HOs with similar biochemical parameters, while AtHO‐2 lacks such an activity [10].

AtHO‐1 is a prototype of plant HOs and has been studied extensively. A long hypocotyl phenotype has been mapped to the *HY1* locus, which harbors the *AtHO‐1* gene and whose mutation causes a deficiency of phytochrome chromophore [11, 12, 13]. Mutation of the *AtHO‐1* gene also generates a genomes uncoupled (gun) phenotype showing perturbed plastid‐to‐nucleus signaling [14, 15]. In addition, it is involved in drought tolerance by modulating the stomatal aperture and possibly acting as a negative regulator of drought stress signaling [16].

Until recently, most structural studies of canonical HOs were focused on mammalian and bacterial HOs [17]. The known structures include human HO‐1 and HO‐2 [18, 19, 20, 21, 22, 23], rat HO‐1 [24, 25, 26, 27], two cyanobacterial HOs from *Synechocystis* sp. PCC 6803, SynHO‐1 and SynHO‐2 [28, 29], and four pathogenic bacterial HOs, HemO from *Neisseria meningitidis* [30], *Pseudomonas aeruginosa* HO [31], HmuO from *Corynebacterium diphtheriae* [32, 33, 34], and *Leptospira interrogans* HO (LiHO) [35]. Mammalian HOs use NADPH‐cytochrome P450 reductase (CPR) as redox partner to obtain electrons from NADPH, and the electron transfer path has been revealed by the complex structures of rat CPR–HO‐1 [36, 37]. Bacterial and plant HOs prefer ferredoxin‐NADP^+^ reductase (FNR) and ferredoxin, but the interaction between these HOs and their redox partner(s) is unclear. The putative ferredoxin‐binding site of SynHO‐1/‐2 has been proposed based on molecular surface analysis [28, 29]. LiHO needs only FNR for the reaction with ferredoxin being dispensable, and a transient FNR–LiHO complex model has been proposed [35].

Very recently, crystal structure of soybean *Glycine max* HO‐1 (GmHO‐1) was reported and the interactions between GmHO‐1 and ferredoxin were characterized [38], offering the first structural insight into the catalytic mechanism of plant HOs. GmHO‐1 has a sequence similarity of 72% with AtHO‐1 [39], which implies that these two HOs are highly structurally conserved. Despite their similarity and the requirement of reduced ferredoxin as the primary redox partner, the structure–function relationship for plant HOs still await further characterization [40, 41]. In addition, the nonenzymatic heme degradation process to produce the biliverdin isomers, that is, a process referred to as coupled oxidation and commonly occurring in heme‐binding proteins such as myoglobin and cytochrome *b*_5_ [42, 43], has not been tested for AtHO‐1. In this work, we characterize the heme‐binding and ‐degrading activities of AtHO‐1, differentiate the coupled oxidation and enzymatic processes, describe the heme–AtHO‐1 structure, and present a previously unreported feature of plant HOs.

## Materials and methods

### Protein expression and purification

2Gene sequence (The Arabidopsis Information Resource database identifier: At2g26670) encoding the mature AtHO‐1 (residues 55–282) was amplified by PCR and then inserted into the Novagen pET‐28a(+) vector between the *Nco* I and *EcoR* I restriction sites. The resulting construct encoded an N‐terminal His tagged AtHO‐1 and was transformed into *Escherichia coli* BL21(DE3) cells. Expression of the recombinant protein was induced by 200 μm of isopropyl β‐d‐thiogalactoside when the cell culture had an optical density of 0.8 at 600 nm. The culture was then grown at 16 °C for 16 h, pelleted, and resuspended in the lysis buffer (200 mm of NaCl and 20 mm of Tris–HCl, pH 7.5) with additional 5 mm of imidazole. Cell resuspension was lysed by sonication in an ice water bath and the debris was removed by centrifugation. The cleared lysate was incubated with nickel nitrilotriacetic acid agarose resin (QIAGEN, Shanghai, China) at 4 °C for 1 h, packed into a column, and washed with the lysis buffer supplemented with 50 mm of imidazole. Recombinant AtHO‐1 was eluted with 200 mm of imidazole in the lysis buffer. For further purification by size‐exclusion chromatography (SEC), the eluate was concentrated by ultrafiltration and then loaded onto a HiLoad 16/60 Superdex 200 column (GE Healthcare, Beijing, China) equilibrated and eluted with the lysis buffer. Peak fractions corresponding to recombinant AtHO‐1 were collected, pooled, and analyzed by SDS/PAGE. Recombinant *Zea mays* ferredoxin from and *A. thaliana* FNR were obtained as previously described [44].

To prepare the heme–AtHO‐1 complex, hemin (ferric chloride heme) was first dissolved in dimethyl sulfoxide to obtain a stock solution, and then hemin and the purified AtHO‐1 were mixed in a molar ratio of 2 : 1. The mixture was incubated at 4 °C for 1 h before being concentrated and applied onto a HiLoad 16/60 Superdex 200 column equilibrated and eluted with the lysis buffer. Peak fractions corresponding to the heme–AtHO‐1 complex were collected, pooled, and concentrated for coupled oxidation assay and crystallization.

### Isothermal titration calorimetry

Isothermal titration calorimetry (ITC) experiment was performed on a MicroCal iTC200‐2 calorimeter (Malvern Panalytical, Westborough, MA, USA) at 25 °C. Hemin was dissolved to a concentration of 20 mm in 0.1 m NaOH, and then diluted ×20 with titration buffer (150 mm of NaCl and 100 mm of Tris–HCl, pH 7.5). The purified AtHO‐1 solution was changed to the titration buffer by ultrafiltration. The titration series consisted of 20 injections of hemin solution (first injection of 0.4 μL and subsequent injections of 2 μL) into the AtHO‐1 solution.

### Coupled oxidation assay

The purified heme–AtHO‐1 complex was diluted to 10 μm for the assay, which was performed using the lysis buffer. Reagents were from Sigma‐Aldrich (St Louis, MO, USA) unless noted. The concentrations used were 3 μm, 0.2 mm, and 0.9 mm for catalase, ascorbate, and deferoxamine (DFO), respectively.

### HO activity assay

Heme oxygenase activity was assayed following a previously described procedure [45]. Unless specified otherwise, the reaction mixture contained 10 μm of AtHO‐1, 2.5 μm of ferredoxin, 2.5 μm of FNR, 3 μm of catalase, 0.9 mm of DFO, 10 μm of hemin, and 400 μm of NADPH. Reaction was started by finally adding NADPH, and spectra were recorded from 350 to 800 nm every 4 min for 40 min.

### Crystallization and structure determination

The purified heme–AtHO‐1 complex was concentrated to 10 mg·mL^−1^ for crystal screen. Crystals were grown with the sitting‐drop vapor‐diffusion method by mixing 1 μL of heme–AtHO‐1 complex with 1 μL of reservoir solution consisting of 0.17 m ammonium acetate, 85 mm sodium acetate trihydrate, pH 4.6, 25.5% (w/v) polyethylene glycol 4000, and 15% (v/v) glycerol at 16 °C. For data collection, crystals were transferred step by step into the reservoir solution containing 15%, 20%, and 25% (v/v) glycol, and then flash‐cooled in liquid nitrogen.

Diffraction data were collected at 100 K at the Shanghai Synchrotron Radiation Facility beamline BL17U1 and processed with the program hkl2000 [46]. The structure was determined by molecular replacement with the program phaser in phenix [47, 48]. The coordinates of *C. diphtheriae* heme oxygenase HmuO (PDB entry 1IW0) [32] were used as the search model. Two AtHO‐1 molecules were found in one asymmetric unit. The model was refined by iterative cycles of manual correction in coot [49] and automatic refinement in phenix [48]. The overall model quality was validated by molprobity [50]. Figures showing the structure were drawn with pymol (Schrödinger, LLC, New York, NY, USA).

## Results

### Purified AtHO‐1 is a mixture of apo‐ and heme‐bound forms

**Fig. 1:**
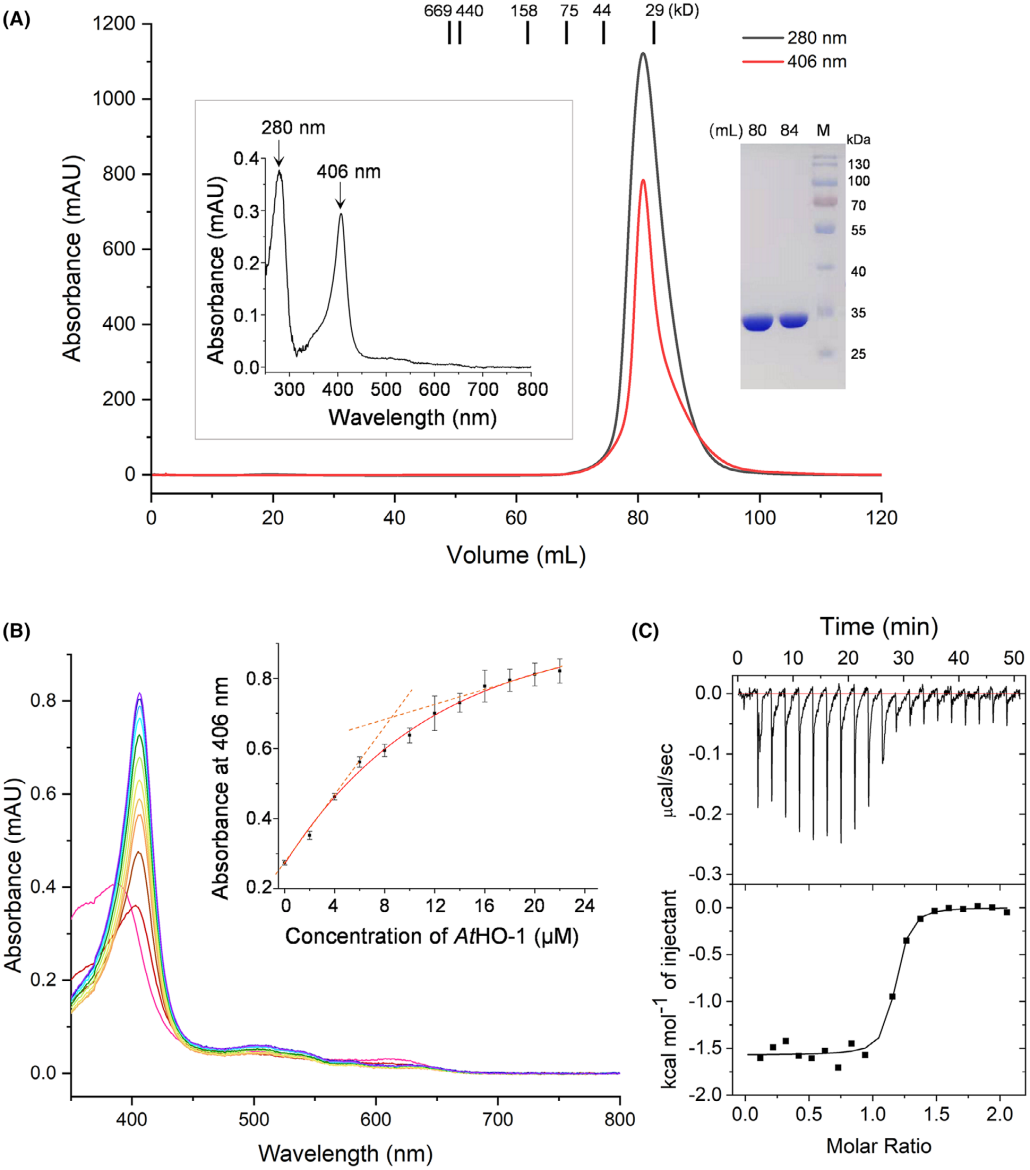
Characterization of purified AtHO‐1 protein. (A) The SEC profile, SDS/PAGE, and absorption spectrum for the purified recombinant AtHO‐1. The absorption spectrum (left inset) was recorded in a wavelength range from 250 to 800 nm. The peak fraction in the SEC profile was subjected to SDS/PAGE (right inset). (B) The absorption spectra of heme with increased concentration of AtHO‐1 (from red to purple). Heme spectrum in the absence of AtHO‐1 is in magenta. Inset: The absorbance difference at 406 nm with increased concentration of AtHO‐1. Error bars represent the standard deviation from three independent measurements. The fitted linear equations are in dashed lines. (C) Isothermal titration of AtHO‐1 with heme. Solid line in the lower panel shows the fit of the integrated heats to one single‐site binding model. [Colour figure can be viewed at wileyonlinelibrary.com]

Recombinant AtHO‐1 purified by SEC has heme absorption maxima in the visible range (Fig. 1A). This indicates that the sample contains heme bound to the protein during its heterologous expression in *E. coli*. To estimate the ratio of unliganded (apo) to heme‐bound AtHO‐1, we titrated the sample to 10 μm heme (Fig. 1B). The Soret maximum has a red shift with gradual increment, confirming formation of the heme–AtHO‐1 complex. It is postulated that the initial rapid increase at 406 nm reflects the amount of heme–AtHO‐1 complex; after saturation of free heme, the increase at 406 nm reflects the heme–AtHO‐1 fraction of the added sample. By linear fitting, we calculated the ratio of apo to heme‐bound AtHO‐1, which was *ca*. 85–15%. The fact that partial AtHO‐1 binds heme during purification suggests that AtHO‐1 possesses a relative high affinity to heme. We then used ITC to quantify the affinity (Fig. 1C), and fitting of the titration curve yielded a submicromolar dissociation constant (*K*_d_ = 0.26 ± 0.01 μm) between AtHO‐1 and heme. This *K*_d_ value is *ca*. 6‐fold lower than reported values based on spectrophotometric titration [10, 40], and the discrepancy could be due to method difference and the fact that the sample used for ITC was a mixture of apo‐ and heme‐bound AtHO‐1.

### Characterization of coupled oxidation

**Fig. 2:**
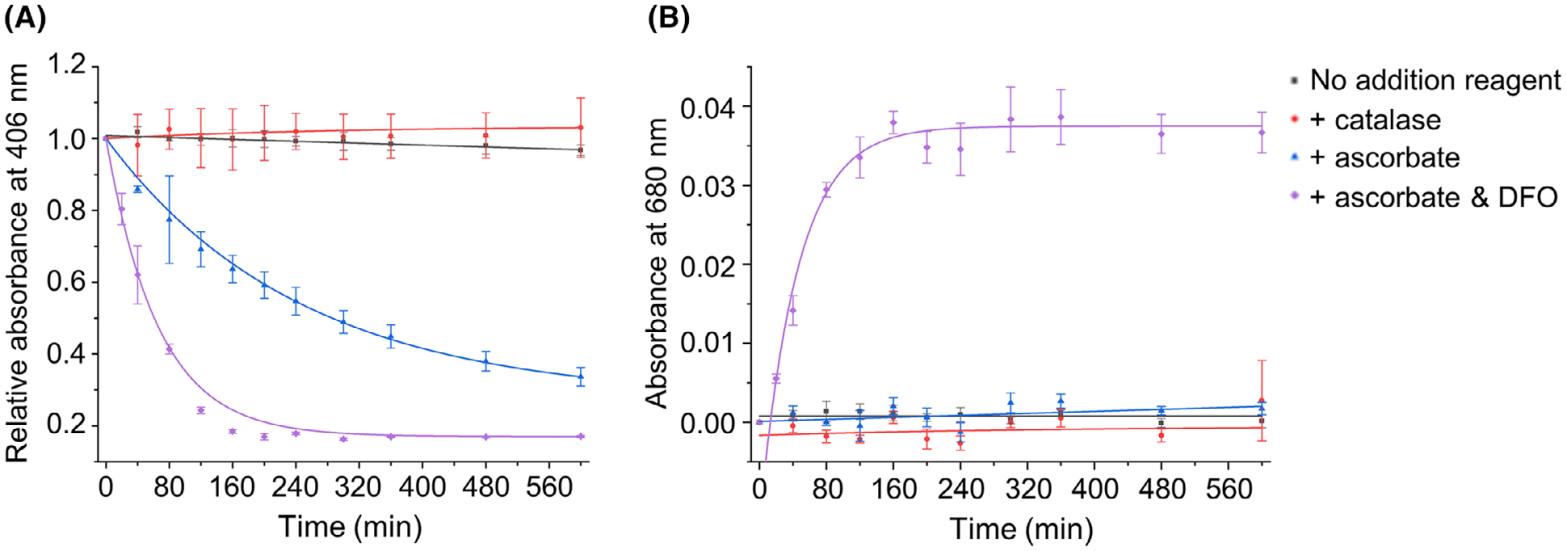
Characterization of coupled oxidation. The absorbance difference at 406 nm of heme–AtHO‐1 (A) and at 680 nm of biliverdin (B). Additions of catalase, ascorbate, and ascorbate with DFO are shown in red, blue, and purple, respectively. Error bars represent the standard deviation from three independent measurements. [Colour figure can be viewed at wileyonlinelibrary.com]

For AtHO‐1 activity characterization, we first tested the effect of ascorbate and catalase to separate coupled oxidation from enzymatic heme oxygenation (Fig. 2 and Fig. S1). The presence of 3 μm of catalase essentially inhibited the coupled oxidation; in the absence of catalase, the oxidation was enhanced by addition of 200 μm of ascorbate. The iron chelator DFO has been found to enhance iron release in human HO‐1 [51] and be required for the full activity of AtHO‐1 [40]. Our results confirmed that DFO is absolutely needed for the production of biliverdin. Therefore, catalase and DFO were included in the enzymatic assays (see below) for testing the reducing systems.

### Ferredoxin is redundant for FNR‐mediated AtHO‐1 activity

**Fig. 3:**
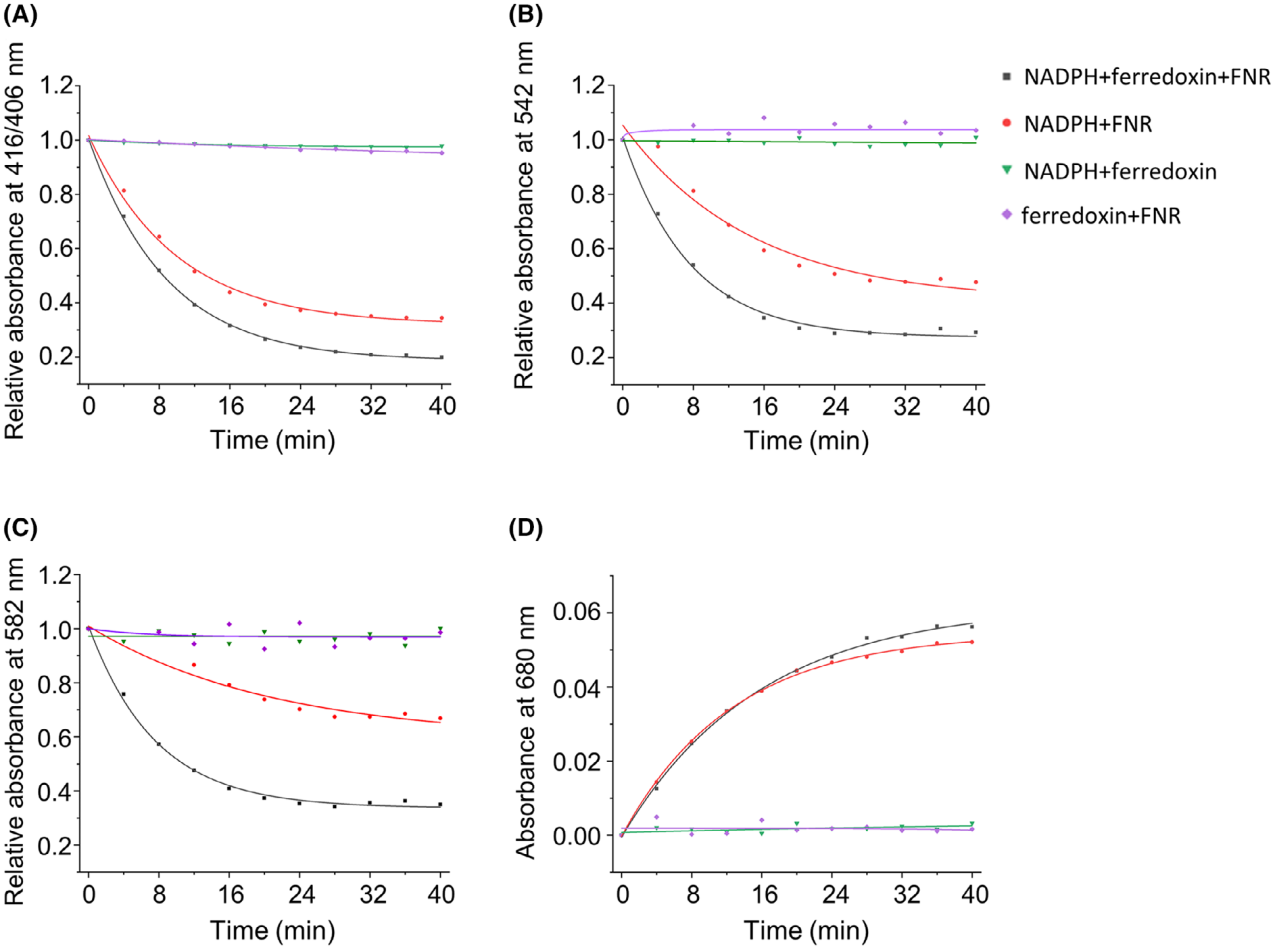
FNR‐dependent AtHO‐1 activity assay. The absorbance difference at the Soret peak (A), 542 nm (B), 582 nm (C), and 680 nm (D). Removals of ferredoxin, FNR, and NADPH are shown in red, green, and purple, respectively. [Colour figure can be viewed at wileyonlinelibrary.com]

The assays were performed in the presence of 3 μm of catalase and 0.9 mm of DFO. The biological reducing system NADPH–FNR–ferredoxin was tested, and the necessity of each component was checked (Fig. 3 and Fig. S2). Absence of ferredoxin only perturbed the HO activity as reflected by changes of the four characteristic absorbance peaks, whereas the absence of NADPH or FNR abolished the activity. These results indicated that ferredoxin is dispensable for the NADPH‐dependent HO activity, suggesting that AtHO‐1 could directly interact with FNR to receive electrons from NADPH.

**Fig. 4:**
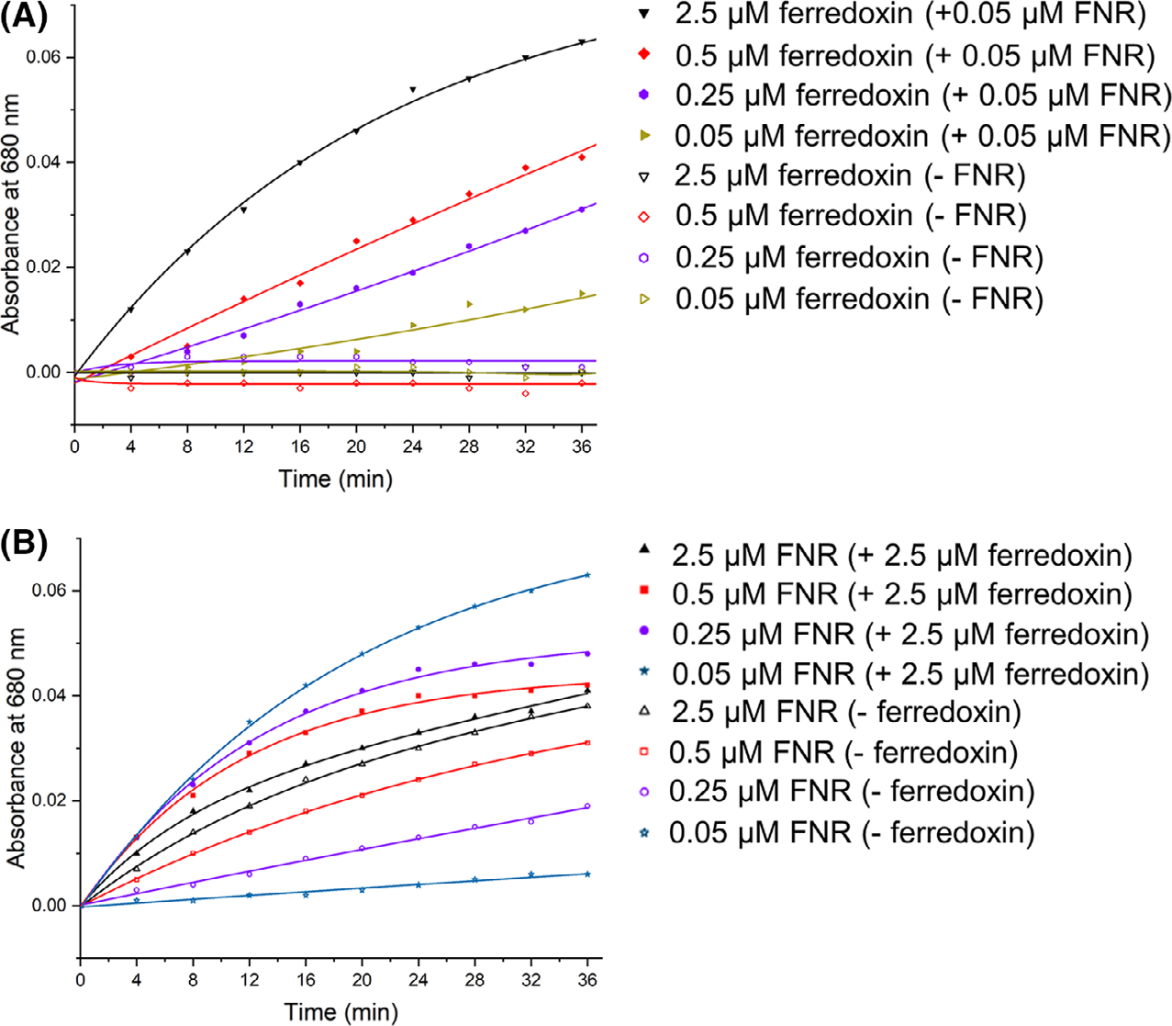
Concentration relationship of FNR and ferredoxin for AtHO‐1 activity. (A) The AtHO‐1 activity assay under different concentrations of ferredoxin in the presence (filled symbols) or absence (open symbols) of FNR. (B) The AtHO‐1 activity assay under different concentrations of FNR in the presence (filled symbols) or absence (open symbols) of ferredoxin. Absorbance at 680 nm was shown and the conditions were labeled. [Colour figure can be viewed at wileyonlinelibrary.com]

To define the role of FNR and ferredoxin for electron transfer, we first tested the concentration dependence of ferredoxin. When the FNR concentration was fixed at 0.05 μm, the activity of AtHO‐1 increased with the ferredoxin concentration from 0.05 to 2.5 μm (Fig. 4A and Fig. S3). As expected, in the absence of FNR, the activity was totally lost regardless of ferredoxin concentration change. We then tested the concentration dependence of FNR, and found that in the absence of ferredoxin, the activity increased with the FNR concentration from 0.05 to 2.5 μm (Fig. 4B and Fig. S4), indicating that FNR alone is capable of transferring electrons to heme. Interestingly, when ferredoxin was fixed at 2.5 μm, the activity decreased with the increasing FNR concentration (Fig. 4B), which suggests a possibility that FNR has low electron transfer efficiency compared with ferredoxin when the two are competing for AtHO‐1.

### AtHO‐1 structure

**Fig. 5:**
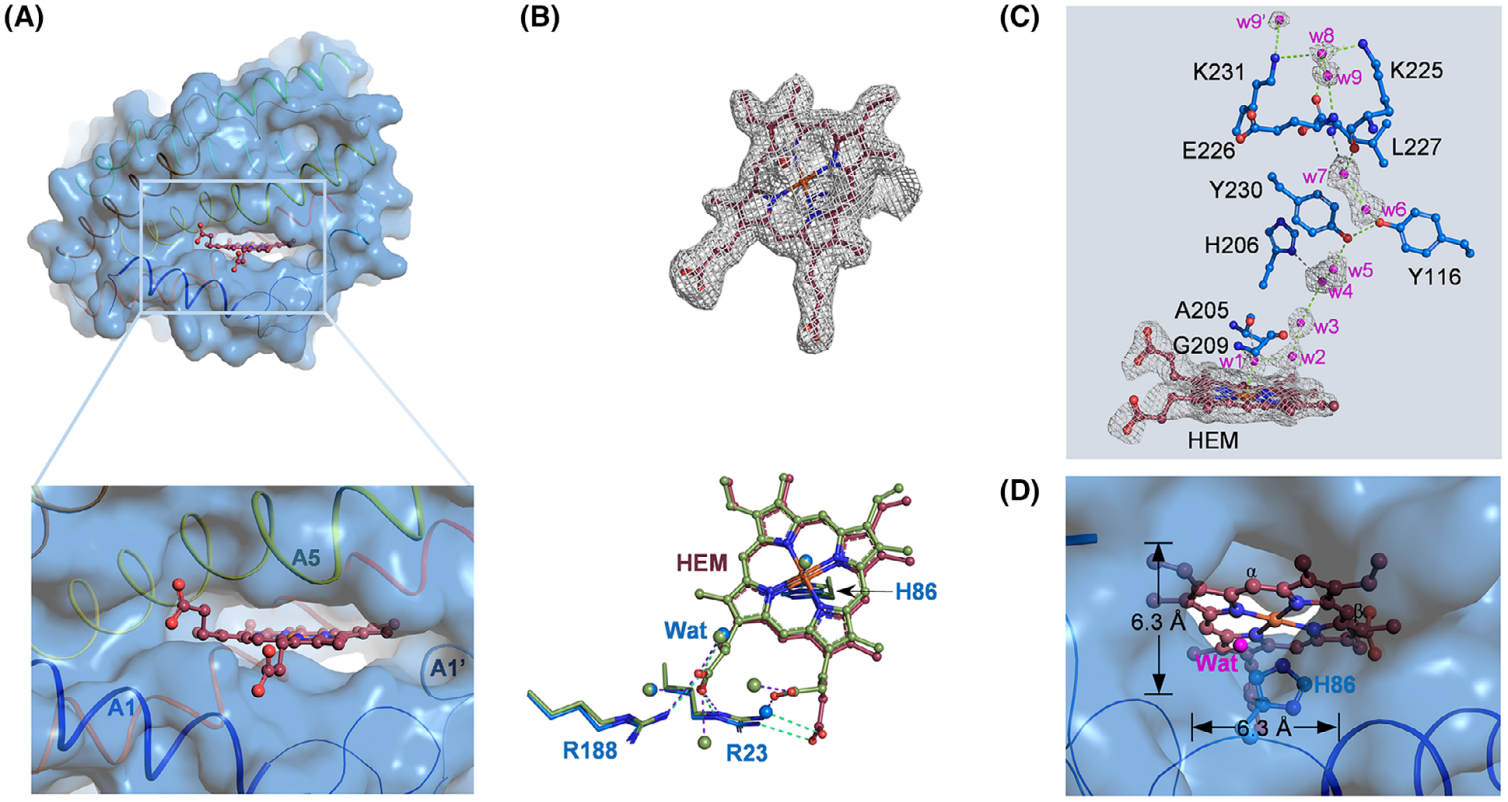
The heme–AtHO‐1 complex structure. (A) Overall structure of heme‐bound AtHO‐1. Protein backbone is in ribbon (colored in blue to red scheme from N‐ to C‐terminus) and the surface is in transparent blue. Heme (brown) is in ball and stick model. Inset shows the heme pocket. (B) Electron density at 1.0σ of heme (upper panel) and comparison with heme–GmHO‐1 (green, lower panel). Residues in AtHO‐1 are shown in blue, and polar interactions between heme and AtHO‐1 are in dashed cyan lines. Interactions between heme and GmHO‐1 are in purple. Water molecules are in spheres. (C) The hydrogen‐bond network in the distal side of heme. Electron density of heme and structured water molecules are shown. (D) The backside of heme pocket. Color scheme same as (A). [Colour figure can be viewed at wileyonlinelibrary.com]

**Fig. 6:**
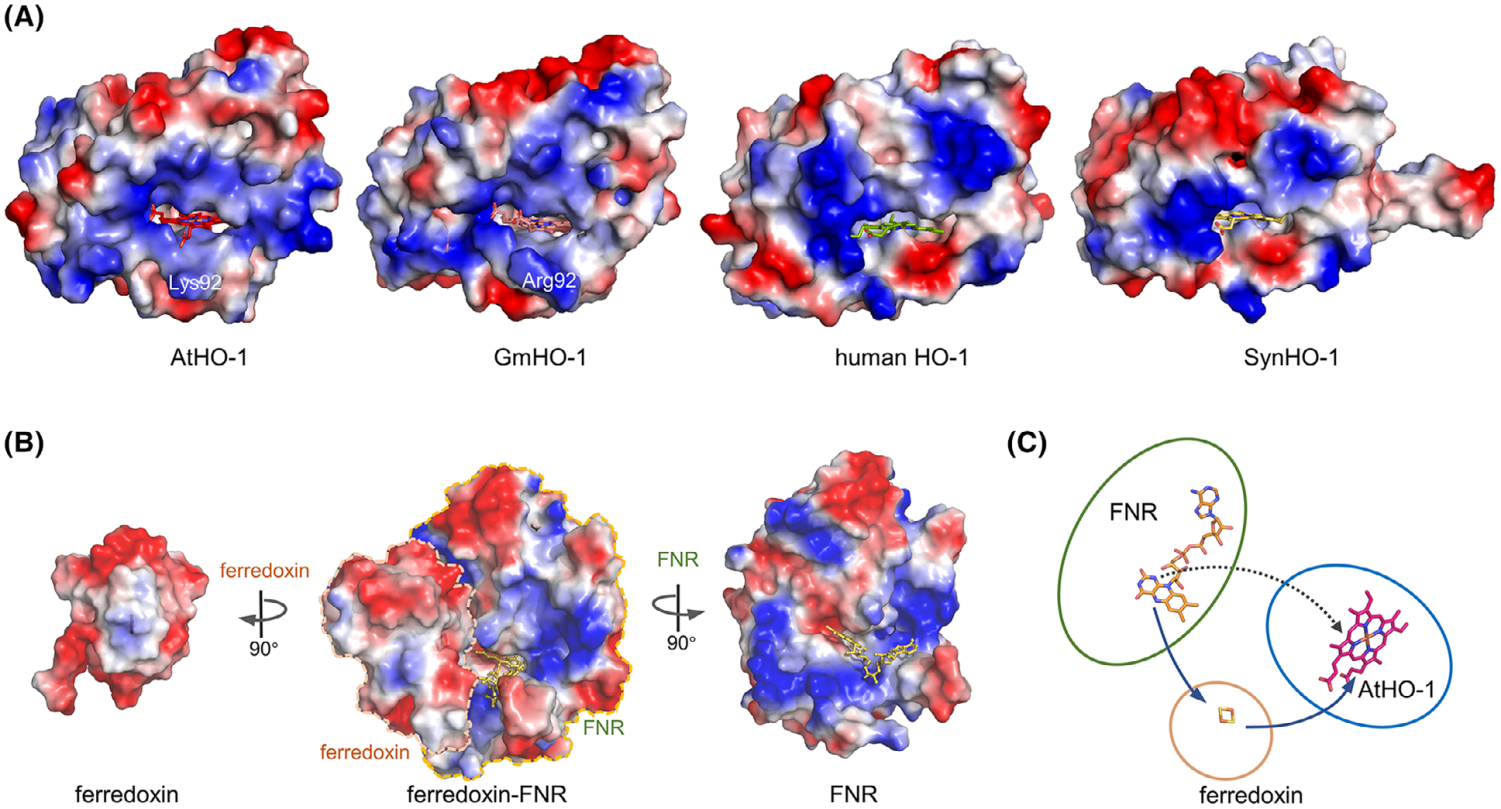
Comparison of HO structures, the FNR–ferredoxin structure, and an electron‐transfer model. (A) Surface potential representation of AtHO‐1, GmHO‐1, human HO‐1, and SynHO‐1. Heme is in stick model, and the position of AtHO‐1 Lys92 and GmHO‐1 Arg92 are indicated. (B) Surface potential of the ferredoxin–FNR complex. The left and right panels show the surface constituting the ferredoxin–FNR interface. (C) Electron‐transfer model. AtHO‐1 is proposed to interact with the FAD pocket to for direct electron transfer (dash line), compared with the canonical electron transfer pathway (solid lines). [Colour figure can be viewed at wileyonlinelibrary.com]

We then set to determine the structure of AtHO‐1, but the attempt to crystalize apo AtHO‐1 was unsuccessful. The heme–AtHO‐1 complex was crystallized and its structure was solved at 2.2‐Å resolution (Table 1). Like in all HOs, heme is located in the pocket between the N‐terminal and fifth helices of AtHO‐1 (Fig. 5A). The heme iron is coordinated by the imidazole group of His86 on the proximal side and a water molecule on the distal side, and the propionate groups are pointing out from the pocket. A conformational difference between AtHO‐1 and GmHO‐1 occurs with respect to the orientation of a propionate group (Fig. 5B). Within the pocket, a hydrogen‐bond network running from the distal water to the protein surface is formed by water molecules and residues including Tyr116, His206, Tyr230, Lys225‐Leu227, and Lys231 (Fig. 5C). On the proximal side, a hole at the backside runs from the α‐meso carbon to the heme pocket (Fig. 5D). Such a hole is also observed in GmHO‐1 structure [38] but not in human HO‐1 or SynHO‐1 (Fig. 6A), confirming the conservation of a specific feature for plant HOs.

**Table 1:** Data collection and structure refinement statistics of heme‐bound AtHO‐1.

PDB	7EQH
Diffraction data	
Resolution (Å)^a^	50.00–2.20 (2.28–2.20)
Space group	P2_1_2_1_2_1_
Wavelength (Å)	0.979
Unit‐cell parameters	
*a*, *b*, *c* (Å)	66.6, 84.8, 92.9
α, β, γ (°)	90, 90, 90
No. of measured reflections	197,033 (19,877)
No. of unique reflections	27,162 (2686)
Completeness (%)	99.8 (100)
Average redundancy	7.3 (7.4)
Mean *I*/σ*I*	14.2 (2.3)
Wilson *B*‐factor (Å^2^)	31.49
*R*_merge_	0.131 (1.000)
*R*_pim_	0.058 (0.587)
CC_1/2_	0.965 (0.850)
Refinement	
Resolution (Å)	34.75–2.20 (2.29–2.20)
No. of reflections used in refinement	25,120 (1647)
No. of reflections used for *R*_free_	1306 (82)
*R*_work_ ^b^ (%)	20.8 (29.4)
*R*_free_ ^c^ (%)	25.0 (35.8)
Number of atoms	
Protein	3504
Ligand	86
Water	165
Average *B*‐factor (Å^2^)	
Protein	35.13
Ligand	30.86
Water	37.40
R.m.s deviations	
Bond length (Å)	0.004
Bond angles (°)	0.65
Ramachandran plot	
Favored (%)	99.05
Allowed (%)	0.95

^a^ Values in parentheses are for highest resolution shell.

^b^ *R*_work_ = ∑¦¦*F*_o_¦ − ¦*F*_c_¦¦/∑¦*F*_o_¦, where *F*_o_ and *F*_c_ are the observed and calculated structure factors, respectively.

^c^ *R*_free_ is the cross‐validated *R* factor computed for a test set of 5% of the reflections, which were omitted during refinement.

## Discussion

In this study, we determined the crystal structure of heme–AtHO‐1 complex at a resolution of 2.2 Å. The surface potentials of AtHO‐1 and GmHO‐1 are highly similar despite differences in a heme propionate group and the exposed residues (Fig. 6A). A major difference is Lys92 in AtHO‐1 and Arg92 in GmHO‐1 (Fig. 6A), while the interactions of heme to HOs are identical (Fig. 5B). In mammalian HO‐1s, the charged surface around the heme pocket was proposed to facilitate the formation of complex with the electron donor NADPH–CPR [36, 37]. For cyanobacterial HOs, the surface is proposed to interact with the physiological oxidative partner, reduced ferredoxin [28, 29]. The [2Fe‐2S] cluster of ferredoxin directly receives electrons from FNR as shown in the FNR–ferredoxin complex structure (Fig. 6B) [52]. The reduced ferredoxin needs to dissociate from FNR to expose the [2Fe‐2S] side because this side is the HO‐interacting site as revealed by NMR titration [38].

The results presented here indicate that ferredoxin is dispensable for AtHO‐1 activity (Figs 3 and 4B). In the *in vitro* assay, reduced ferredoxin was obtained by supplying FNR and NADPH, and when ferredoxin was absent, the reaction still proceeded. Thus, FNR alone could transfer electrons to heme–AtHO‐1, although the process was slow. Addition of ferredoxin clearly enhanced HO reaction by promoting the efficient electron transfer to heme–AtHO‐1. This does not conflict with past assays in which the reduced ferredoxin was generated by light irradiation of isolated thylakoid membranes [40]. Because ferredoxin is abundant in chloroplast, the FNR–ferredoxin system should be the major *in‐vivo* electron donor for AtHO‐1. We conclude that electrons can also be directly transferred from FNR to AtHO‐1. This scenario has been observed in LiHO, which receives electrons from FNR via a possible pathway on the distal side [35]. Interestingly, the FNR in the aerobic spirochete *L. interrogans* is a close relative of plastid‐type FNR in eukaryotes. The hydrogen‐bond network observed in AtHO‐1 on the distal side allows a possibility for an electron pathway directly linking the flavin coenzyme to the substrate heme (Fig. 6C). The biological implication that AtHO‐1 catalysis can bypass ferredoxin and be solely dependent on FNR awaits to be tested *in vivo*.

## Conflict of interest

The authors declare no conflict of interest.

## Author contributions

JW, XL, and LL conceived and designed the project, JW, XL, J‐WC, TY, and YM acquired the data, XW and LL analyzed and interpreted the data, JW and LL wrote the paper.

## Supporting information


**Fig. S1.** Spectra for characterization of coupled oxidation, related to Fig. 2. The assays were performed without additional reagents (A), with catalase (B), with ascorbate (C), and with ascorbate and DFO (D).

**Fig. S2.** Spectra for characterization of FNR‐dependent AtHO‐1 activity assay, related to Fig. 3. The assays were performed with NADPH, ferredoxin, and FNR (A), with NADPH and FNR (B), with NADPH and ferredoxin (C), and with ferredoxin and FNR (D).

**Fig. S3.** Spectra of the concentration dependence for ferredoxin, related to Fig. 4A. The concentration of ferredoxin ranges from 2.5 to 0.05 μM from top to bottom, (A) at fixed FNR concentration of 0.05 μM, (B) in absence of FNR.

**Fig. S4.** Spectra of the concentration dependence for FNR, related to Fig. 4B. The concentration of FNR ranges from 2.5 to 0.05 μM from top to bottom, (A) at fixed ferredoxin concentration of 0.05 μM, (B) in absence of ferredoxin.

## Data Availability

The atomic coordinates and structure factors (code 7EQH) have been deposited in the Protein Data Bank (http://wwpdb.org/).

## References

[1] Wilks A . Heme oxygenase: evolution, structure, and mechanism. Antioxid Redox Signal. 2002;4(4):603–14. 12230872 10.1089/15230860260220102

[2] Unno M , Matsui T , Ikeda‐Saito M . Structure and catalytic mechanism of heme oxygenase. Nat Prod Rep. 2007;24(3):553–70. 17534530 10.1039/b604180a

[3] Terry MJ , Linley PJ , Kohchi T . Making light of it: the role of plant haem oxygenases in phytochrome chromophore synthesis. Biochem Soc Trans. 2002;30(4):604–9. 12196146 10.1042/

[4] Mochizuki N , Tanaka R , Grimm B , Masuda T , Moulin M , Smith AG , et al. The cell biology of tetrapyrroles: a life and death struggle. Trends Plant Sci. 2010;15(9):488–98. 20598625 10.1016/j.tplants.2010.05.012

[5] Bryant DA , Hunter CN , Warren MJ . Biosynthesis of the modified tetrapyrroles—the pigments of life. J Biol Chem. 2020;295(20):6888–925. 32241908 10.1074/jbc.REV120.006194 PMC7242693

[6] Shekhawat GS , Verma K . Haem oxygenase (HO): an overlooked enzyme of plant metabolism and defence. J Exp Bot. 2010;61(9):2255–70. 20378668 10.1093/jxb/erq074

[7] Shimizu T , Huang D , Yan F , Stranava M , Bartosova M , Fojtíková V , et al. Gaseous O_2_, NO, and CO in signal transduction: structure and function relationships of heme‐based gas sensors and heme‐redox sensors. Chem Rev. 2015;115(13):6491–533. 26021768 10.1021/acs.chemrev.5b00018

[8] Davis SJ , Bhoo SH , Durski AM , Walker JM , Vierstra RD . The heme‐oxygenase family required for phytochrome chromophore biosynthesis is necessary for proper photomorphogenesis in higher plants. Plant Physiol. 2001;126(2):656–69. 11402195 10.1104/pp.126.2.656 PMC111157

[9] Emborg TJ , Walker JM , Noh B , Vierstra RD . Multiple heme oxygenase family members contribute to the biosynthesis of the phytochrome chromophore in Arabidopsis. Plant Physiol. 2006;140(3):856–68. 16428602 10.1104/pp.105.074211 PMC1400562

[10] Gisk B , Yasui Y , Kohchi T , Frankenberg‐Dinkel N . Characterization of the haem oxygenase protein family in *Arabidopsis thaliana* reveals a diversity of functions. Biochem J. 2010;425(2):425–34. 19860740 10.1042/BJ20090775

[11] Parks BM , Quail PH . Phytochrome‐deficient hy1 and hy2 long hypocotyl mutants of Arabidopsis are defective in phytochrome chromophore biosynthesis. Plant Cell. 1991;3(11):1177–86. 12324588 10.1105/tpc.3.11.1177 PMC160084

[12] Muramoto T , Kohchi T , Yokota A , Hwang I , Goodman HM . The Arabidopsis photomorphogenic mutant hy1 is deficient in phytochrome chromophore biosynthesis as a result of a mutation in a plastid heme oxygenase. Plant Cell. 1999;11(3):335–48. 10072395 10.1105/tpc.11.3.335 PMC144190

[13] Davis SJ , Kurepa J , Vierstra R . The *Arabidopsis thaliana* HY1 locus, required for phytochrome‐chromophore biosynthesis, encodes a protein related to heme oxygenases. Proc Natl Acad Sci USA. 1999;96(11):6541–6. 10339624 10.1073/pnas.96.11.6541 PMC26918

[14] Susek RE , Ausubel FM , Chory J . Signal transduction mutants of Arabidopsis uncouple nuclear CAB and RBCS gene expression from chloroplast development. Cell. 1993;74(5):787–99. 7690685 10.1016/0092-8674(93)90459-4

[15] Mochizuki N , Brusslan JA , Larkin R , Nagatani A , Chory J . Arabidopsis genomes uncoupled 5 (GUN5) mutant reveals the involvement of Mg‐chelatase H subunit in plastid‐to‐nucleus signal transduction. Proc Natl Acad Sci USA. 2001;98(4):2053–8. 11172074 10.1073/pnas.98.4.2053 PMC29380

[16] Xie Y , Mao Y , Duan X , Zhou H , Lai D , Zhang Y , et al. Arabidopsis HY1‐modulated stomatal movement: an integrative hub is functionally associated with ABI4 in dehydration‐induced ABA responsiveness. Plant Physiol. 2016;170(3):1699–713. 26704641 10.1104/pp.15.01550 PMC4775125

[17] Sugishima M , Wada K , Fukuyama K . Recent advances in the understanding of the reaction chemistries of the heme catabolizing enzymes HO and BVR based on high resolution protein structures. Curr Med Chem. 2020;27(21):3499–518. 30556496 10.2174/0929867326666181217142715 PMC7509768

[18] Schuller DJ , Wilks A , Ortiz de Montellano PR , Poulos TL . Crystal structure of human heme oxygenase‐1. Nat Struct Biol. 1999;6(9):860–7. 10467099 10.1038/12319

[19] Lad L , Schuller DJ , Shimizu H , Friedman J , Li H , Ortiz de Montellano PR , et al. Comparison of the heme‐free and ‐bound crystal structures of human heme oxygenase‐1. J Biol Chem. 2003;278(10):7834–43. 12500973 10.1074/jbc.M211450200

[20] Lad L , Wang J , Li H , Friedman J , Bhaskar B , Ortiz de Montellano PR , et al. Crystal structures of the ferric, ferrous, and ferrous‐NO forms of the Asp140Ala mutant of human heme oxygenase‐1: catalytic implications. J Mol Biol. 2003;330(3):527–38. 12842469 10.1016/s0022-2836(03)00578-3

[21] Wang J , Niemevz F , Lad L , Huang L , Alvarez DE , Buldain G , et al. Human heme oxygenase oxidation of 5‐ and 15‐phenylhemes. J Biol Chem. 2004;279(41):42593–604. 15297453 10.1074/jbc.M406346200

[22] Lad L , Friedman J , Li H , Bhaskar B , Ortiz de Montellano PR , Poulos TL . Crystal structure of human heme oxygenase‐1 in a complex with biliverdin. Biochemistry. 2004;43(13):3793–801. 15049686 10.1021/bi035451l

[23] Bianchetti CM , Yi L , Ragsdale SW , Phillips GN Jr . Comparison of apo‐ and heme‐bound crystal structures of a truncated human heme oxygenase‐2. J Biol Chem. 2007;282(52):37624–31. 17965015 10.1074/jbc.M707396200 PMC2896506

[24] Sugishima M , Omata Y , Kakuta Y , Sakamoto H , Noguchi M , Fukuyama K . Crystal structure of rat heme oxygenase‐1 in complex with heme. FEBS Lett. 2000;471(1):61–6. 10760513 10.1016/s0014-5793(00)01353-3

[25] Sugishima M , Sakamoto H , Kakuta Y , Omata Y , Hayashi S , Noguchi M , et al. Crystal structure of rat apo‐heme oxygenase‐1 (HO‐1): mechanism of heme binding in HO‐1 inferred from structural comparison of the apo and heme complex forms. Biochemistry. 2002;41(23):7293–300. 12044160 10.1021/bi025662a

[26] Sugishima M , Sakamoto H , Higashimoto Y , Noguchi M , Fukuyama K . Crystal structure of rat heme oxygenase‐1 in complex with biliverdin‐iron chelate. Conformational change of the distal helix during the heme cleavage reaction. J Biol Chem. 2003;278(34):32352–8. 12794075 10.1074/jbc.M303682200

[27] Sato H , Sugishima M , Sakamoto H , Higashimoto Y , Shimokawa C , Fukuyama K , et al. Crystal structure of rat haem oxygenase‐1 in complex with ferrous verdohaem: presence of a hydrogen‐bond network on the distal side. Biochem J. 2009;419(2):339–45. 19154182 10.1042/BJ20082279

[28] Sugishima M , Migita CT , Zhang X , Yoshida T , Fukuyama K . Crystal structure of heme oxygenase‐1 from cyanobacterium *Synechocystis* sp. PCC 6803 in complex with heme. Eur J Biochem. 2004;271(22):4517–25. 15560792 10.1111/j.1432-1033.2004.04411.x

[29] Sugishima M , Hagiwara Y , Zhang X , Yoshida T , Migita CT , Fukuyama K . Crystal structure of dimeric heme oxygenase‐2 from *Synechocystis* sp. PCC 6803 in complex with heme. Biochemistry. 2005;44(11):4257–66. 15766254 10.1021/bi0480483

[30] Schuller DJ , Zhu W , Stojiljkovic I , Wilks A , Poulos TL . Crystal structure of heme oxygenase from the Gram‐negative pathogen *Neisseria meningitidis* and a comparison with mammalian heme oxygenase‐1. Biochemistry. 2001;40(38):11552–8. 11560504 10.1021/bi0110239

[31] Friedman J , Lad L , Li H , Wilks A , Poulos TL . Structural basis for novel δ‐regioselective heme oxygenation in the opportunistic pathogen *Pseudomonas aeruginosa* . Biochemistry. 2004;43(18):5239–45. 15122889 10.1021/bi049687g

[32] Hirotsu S , Chu GC , Unno M , Lee DS , Yoshida T , Park SY , et al. The crystal structures of the ferric and ferrous forms of the heme complex of HmuO, a heme oxygenase of *Corynebacterium diphtheriae* . J Biol Chem. 2004;279(12):11937–47. 14645223 10.1074/jbc.M311631200

[33] Unno M , Matsui T , Chu GC , Couture M , Yoshida T , Rousseau DL , et al. Crystal structure of the dioxygen‐bound heme oxygenase from *Corynebacterium diphtheriae*: implications for heme oxygenase function. J Biol Chem. 2004;279(20):21055–61. 14966119 10.1074/jbc.M400491200

[34] Unno M , Ardèvol A , Rovira C , Ikeda‐Saito M . Structures of the substrate‐free and product‐bound forms of HmuO, a heme oxygenase from *Corynebacterium diphtheriae*: X‐ray crystallography and molecular dynamics investigation. J Biol Chem. 2013;288(48):34443–58. 24106279 10.1074/jbc.M113.486936 PMC3843059

[35] Soldano A , Klinke S , Otero LH , Rivera M , Catalano‐Dupuy DL , Ceccarelli EA . Structural and mutational analyses of the *Leptospira interrogans* virulence‐related heme oxygenase provide insights into its catalytic mechanism. PLoS One. 2017;12(8):e0182535. 28771589 10.1371/journal.pone.0182535 PMC5542595

[36] Sugishima M , Sato H , Higashimoto Y , Harada J , Wada K , Fukuyama K , et al. Structural basis for the electron transfer from an open form of NADPH‐cytochrome P450 oxidoreductase to heme oxygenase. Proc Natl Acad Sci USA. 2014;111(7):2524–9. 24550278 10.1073/pnas.1322034111 PMC3932878

[37] Sugishima M , Sato H , Wada K , Yamamoto K . Crystal structure of a NADPH‐cytochrome P450 oxidoreductase (CYPOR) and heme oxygenase 1 fusion protein implies a conformational change in CYPOR upon NADPH/NADP^+^ binding. FEBS Lett. 2019;593(8):868–75. 30883732 10.1002/1873-3468.13360

[38] Tohda R , Tanaka H , Mutoh R , Zhang X , Lee YH , Konuma T , et al. Crystal structure of higher plant heme oxygenase‐1 and its mechanism of interaction with ferredoxin. J Biol Chem. 2021;296:100217. 33839679 10.1074/jbc.RA120.016271 PMC7948506

[39] Gohya T , Zhang X , Yoshida T , Migita CT . Spectroscopic characterization of a higher plant heme oxygenase isoform‐1 from *Glycine max* (soybean)—coordination structure of the heme complex and catabolism of heme. FEBS J. 2006;273(23):5384–99. 17076701 10.1111/j.1742-4658.2006.05531.x

[40] Muramoto T , Tsurui N , Terry MJ , Yokota A , Kohchi T . Expression and biochemical properties of a ferredoxin‐dependent heme oxygenase required for phytochrome chromophore synthesis. Plant Physiol. 2002;130(4):1958–66. 12481078 10.1104/pp.008128 PMC166706

[41] Linley PJ , Landsberger M , Kohchi T , Cooper JB , Terry MJ . The molecular basis of heme oxygenase deficiency in the pcd1 mutant of pea. FEBS J. 2006;273(12):2594–606. 16817889 10.1111/j.1742-4658.2006.05264.x

[42] Bonnett R , McDonagh AF . The meso‐reactivity of porphyrins and related compounds. VI. Oxidative cleavage of the haem system. The four isomeric biliverdins of the IX series. J Chem Soc Perkin I. 1973;9:881–8. 10.1039/p19730000881 4735584

[43] Wilks A , Ikeda‐Saito M . Heme utilization by pathogenic bacteria: not all pathways lead to biliverdin. Acc Chem Res. 2014;47(8):2291–8. 24873177 10.1021/ar500028n PMC4139177

[44] Wang X , Liu L . Crystal structure and catalytic mechanism of 7‐hydroxymethyl chlorophyll a reductase. J Biol Chem. 2016;291(25):13349–59. 27072131 10.1074/jbc.M116.720342 PMC4933244

[45] Wang J , Guo Q , Li X , Wang X , Liu L . The Arabidopsis locus AT3G03890 encodes a dimeric β‐barrel protein implicated in heme degradation. Biochem J. 2020;477(24):4785–96. 10.1042/BCJ20200712 33284325

[46] Otwinowski Z , Minor W . Processing of X‐ray diffraction data collected in oscillation mode. Methods Enzymol. 1997;276:307–26. 10.1016/S0076-6879(97)76066-X 27754618

[47] McCoy AJ , Grosse‐Kunstleve RW , Adams PD , Winn MD , Storoni LC , Read RJ . Phaser crystallographic software. J Appl Cryst. 2007;40(Pt 4):658–74. 19461840 10.1107/S0021889807021206 PMC2483472

[48] Liebschner D , Afonine PV , Baker ML , Bunkóczi G , Chen VB , Croll TI , et al. Macromolecular structure determination using X‐rays, neutrons and electrons: recent developments in Phenix. Acta Crystallogr D Struct Biol. 2019;75(Pt 10):861–77. 31588918 10.1107/S2059798319011471 PMC6778852

[49] Emsley P , Lohkamp B , Scott WG , Cowtan K . Features and development of Coot. Acta Crystallogr D Biol Crystallogr. 2010;66(Pt 4):486–501. 20383002 10.1107/S0907444910007493 PMC2852313

[50] Williams CJ , Headd JJ , Moriarty NW , Prisant MG , Videau LL , Deis LN , et al. MolProbity: more and better reference data for improved all‐atom structure validation. Protein Sci. 2018;27(1):293–315. 29067766 10.1002/pro.3330 PMC5734394

[51] Liu Y , Ortiz de Montellano PR . Reaction intermediates and single turnover rate constants for the oxidation of heme by human heme oxygenase‐1. J Biol Chem. 2000;275(8):5297–307. 10681502 10.1074/jbc.275.8.5297

[52] Kurisu G , Kusunoki M , Katoh E , Yamazaki T , Teshima K , Onda Y , et al. Structure of the electron transfer complex between ferredoxin and ferredoxin‐NADP^+^ reductase. Nat Struct Biol. 2001;8(2):117–21. 11175898 10.1038/84097

