# Supplementary figures and images for: Enzymological and structural characterization of Arabidopsis thaliana heme oxygenase‐1
Source: FEBS Open Bio. 2022 Jun 20;12(9):1677–87. doi: 10.1002/2211-5463.13453 (PMC9433822; doi:10.1002/2211-5463.13453)

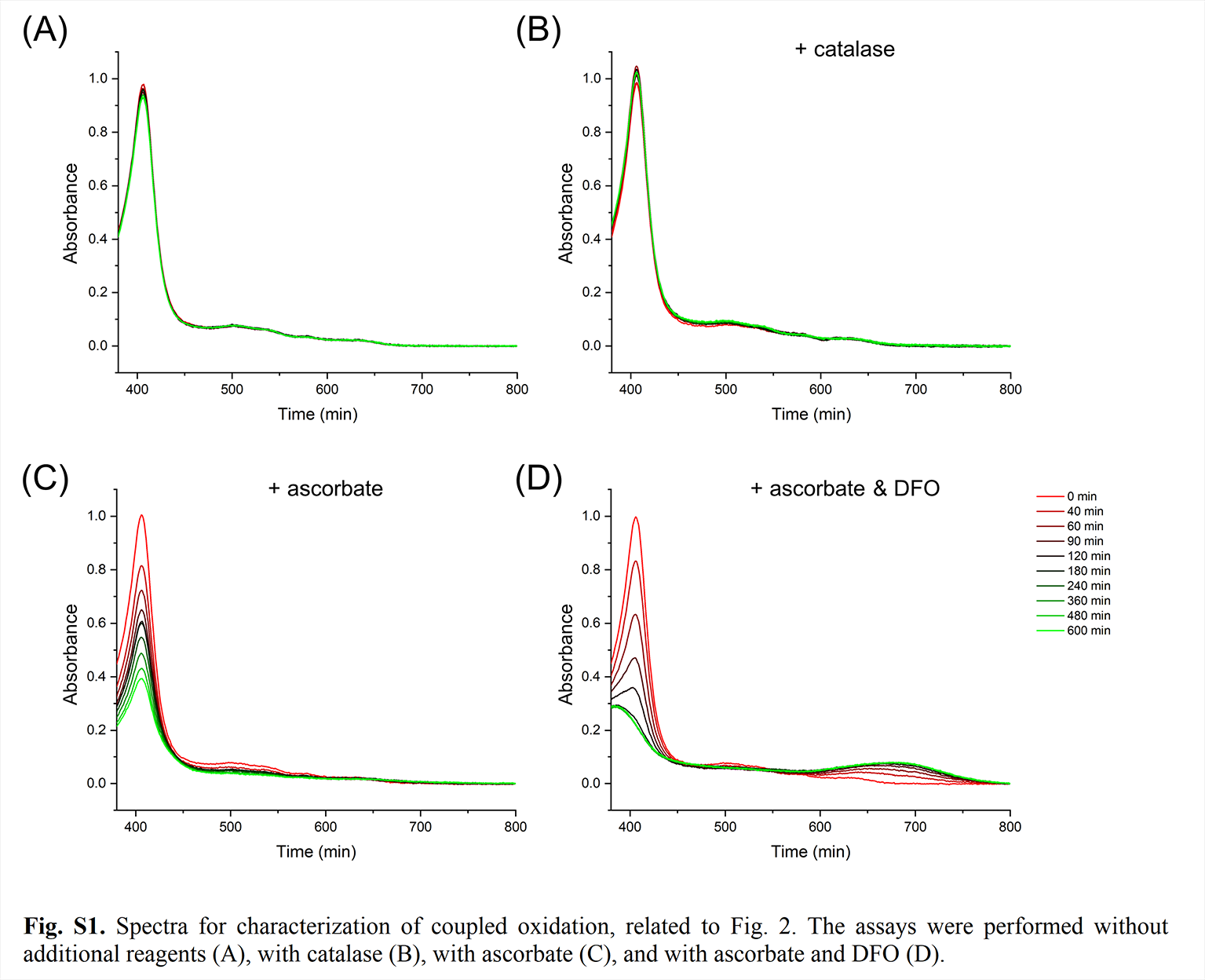

Supplement: Supplementary file 1 — Fig. S1. Spectra for characterization of coupled oxidation, related to Fig. 2. The assays were performed without additional reagents (A), with catalase (B), with ascorbate (C), and with ascorbate and DFO (D). [file FEB4-12-1677-s001.tif]

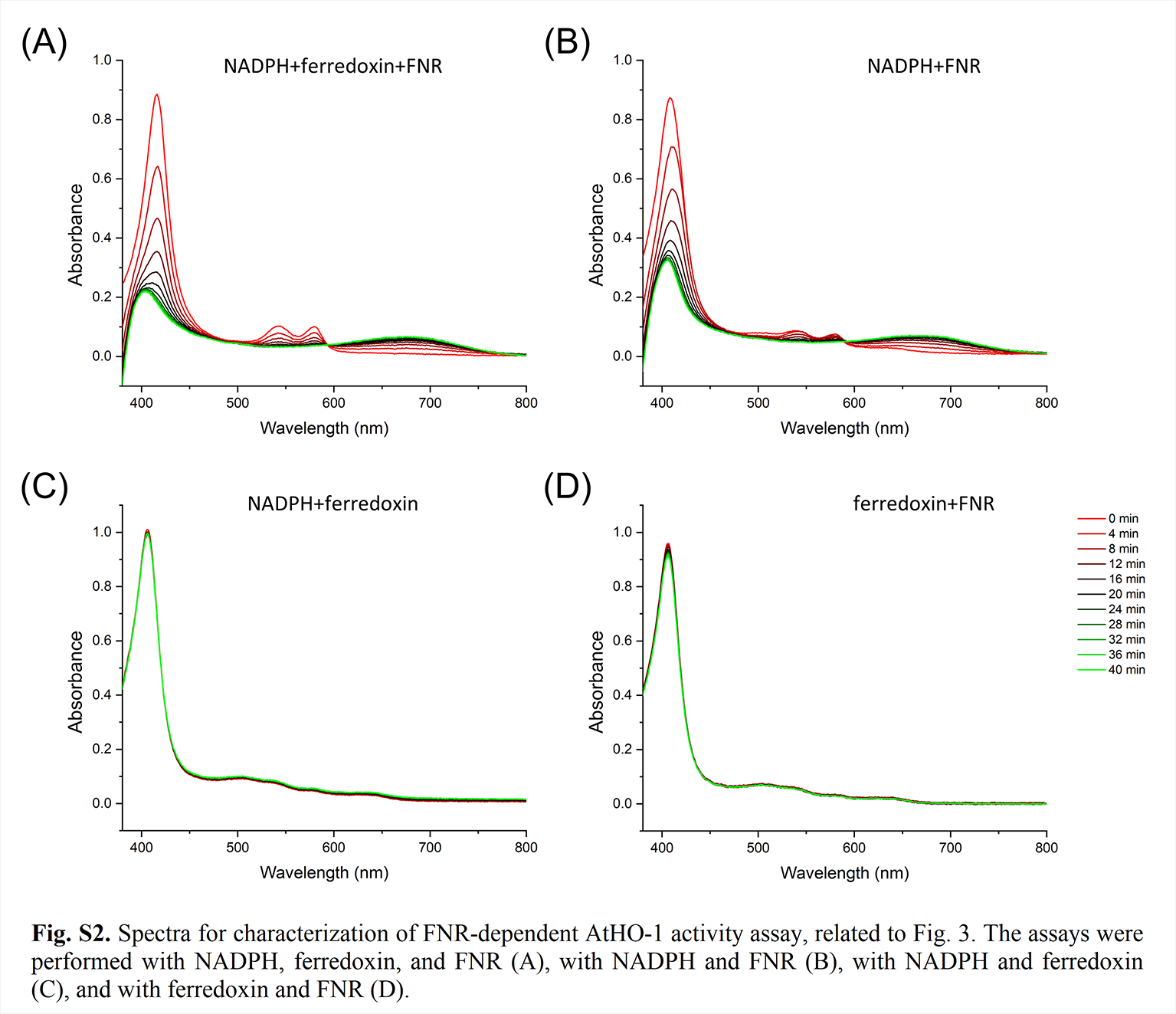

Supplement: Supplementary file 2 — Fig. S2. Spectra for characterization of FNR‐dependent AtHO‐1 activity assay, related to Fig. 3. The assays were performed with NADPH, ferredoxin, and FNR (A), with NADPH and FNR (B), with NADPH and ferredoxin (C), and with ferredoxin and FNR (D). [file FEB4-12-1677-s004.tif]

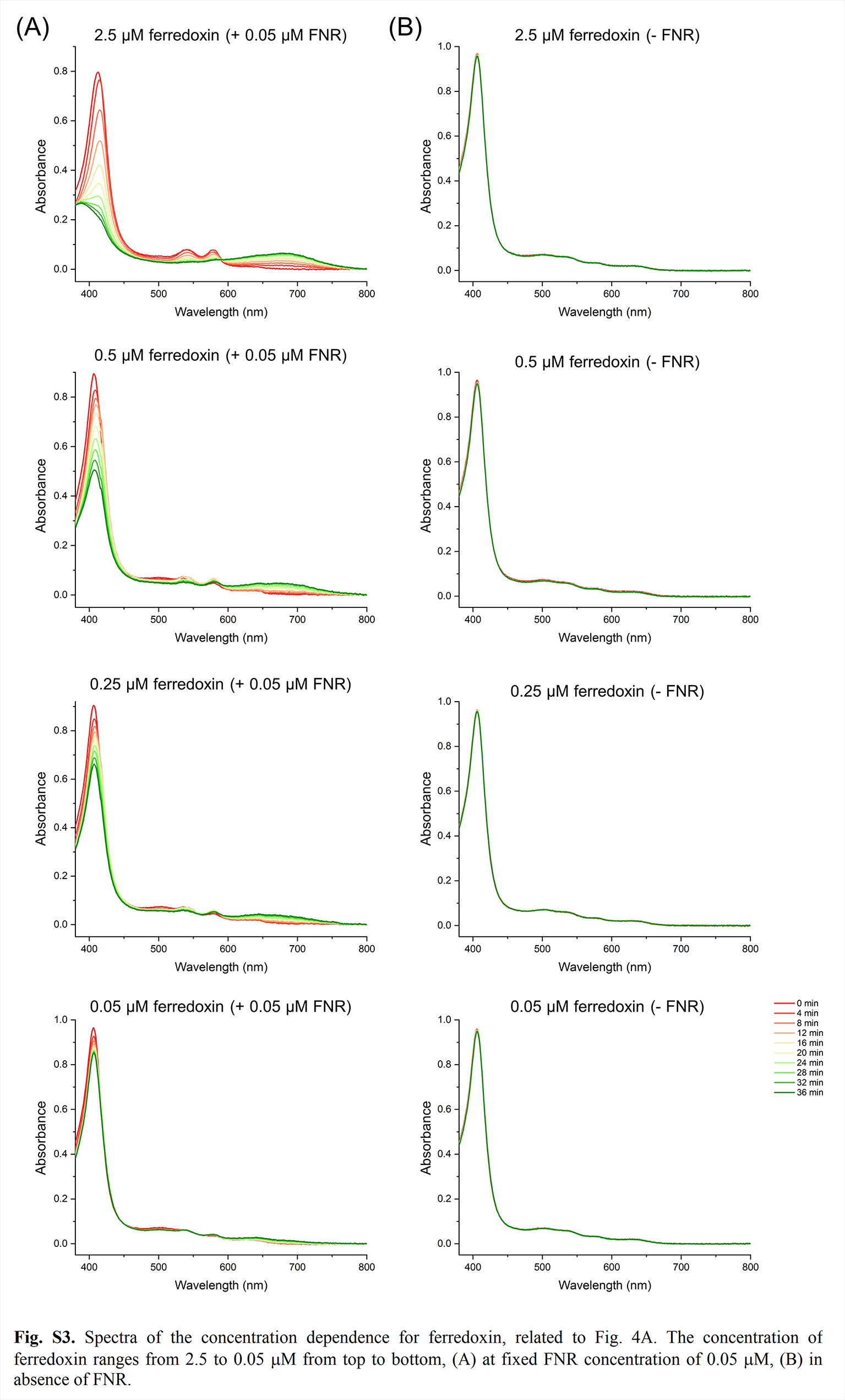

Supplement: Supplementary file 3 — Fig. S3. Spectra of the concentration dependence for ferredoxin, related to Fig. 4A. The concentration of ferredoxin ranges from 2.5 to 0.05 μM from top to bottom, (A) at fixed FNR concentration of 0.05 μM, (B) in absence of FNR. [file FEB4-12-1677-s003.tif]

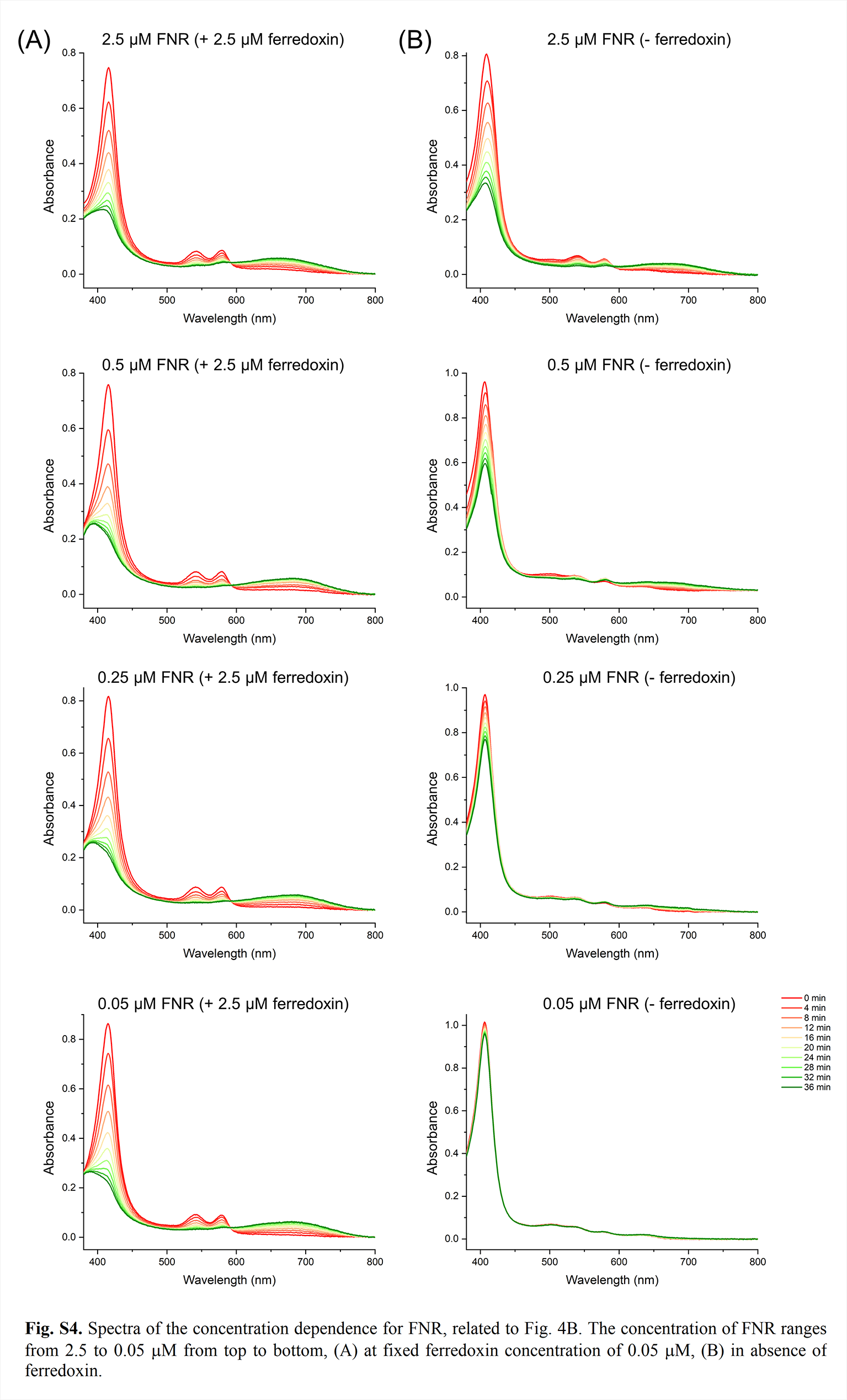

Supplement: Supplementary file 4 — Fig. S4. Spectra of the concentration dependence for FNR, related to Fig. 4B. The concentration of FNR ranges from 2.5 to 0.05 μM from top to bottom, (A) at fixed ferredoxin concentration of 0.05 μM, (B) in absence of ferredoxin. [file FEB4-12-1677-s002.tif]
